# Association of CYP24A1 gene polymorphism with colorectal cancer in the Jiamusi population

**DOI:** 10.1371/journal.pone.0253474

**Published:** 2021-06-30

**Authors:** Lin Chai, Jian Ni, Xiaolin Ni, Nan Zhang, Yang Liu, Zhiwu Ji, Xingwang Zhao, Xiaowen Zhu, Bin Zhao, Guorong Xin, Yu Wang, Fan Yang, Liang Sun, Xiaoquan Zhu, Wenhua Bao, Xiaofang Shui, Fengling Wang, Fujun Chen, Ze Yang

**Affiliations:** 1 The First Affiliated Hospital of Jiamusi University, Heilongjiang, P.R. China; 2 The Key Laboratory of Geriatrics, Beijing Institute of Geriatrics, Beijing Hospital, National Center of Gerontology, National Health Commission, Beijing, P.R. China; 3 Institute of Geriatric Medicine, Chinese Academy of Medical Sciences, Beijing, P.R. China; 4 Graduate School of Chinese Academy of Medical Science and Peking Union Medical College, Beijing, P.R. China; Unicamillus, Saint Camillus International University of Health Sciences, ITALY

## Abstract

**Background:**

The population in Jiamusi has been reported to have the highest prevalence of colorectal cancer (CRC) in China. The genetic causal-effect for this occurrence among the residents remains unclear. Given the long cold seasons with people wearing more clothes and reduced UV exposure, we aimed to study the association between the vitamin D metabolism-related gene CYP24A1 polymorphism and CRC susceptibility.

**Method:**

A case-control study was conducted that included 168 patients with CRC and 710 age-matched healthy individuals as the control group. Plausible susceptible variations were sought and clinical phenotypic-genotype association analysis was performed.

**Results:**

Overall, two CYP24A1 polymorphisms, rs6013905 AX (P = 0.02, OR = 1.89, 95%CI: 1.09–3.29) and rs2762939 GX (P = 0.02, OR = 1.52, 95%CI: 1.08–2.13) were significantly associated with CRC in the Jiamusi population. In the female group, three CYP24A1 polymorphisms, rs6013905 AX (P = 0.04, OR = 2.59, 95%CI: 1.03–6.49), rs2762939 GX (P = 0.01, OR = 2.35, 95%CI: 1.25–4.42), and rs6068816 GG (P = 0.05, OR = 1.89, 95%CI: 0.99–3.59) carriers were significantly associated with CRC. In clinical phenotypic-genotype analysis, rs6013905 GG (P = 0.05, OR = 4.00, 95%CI: 0.92–17.48) and rs2762939 GX (P = 0.03, OR = 4.87, 95%CI: 1.00–23.69) carriers were significantly associated with poorly differentiated CRC, while CYP24A1 rs6068816 AX was significantly associated with the tumor type (P = 0.02, OR = 2.08, 95%CI: 1.10–3.96) and location (P = 0.04, OR = 2.24, 95%CI: 1.05–4.77).

**Conclusion:**

CYP24A1 gene polymorphism may be a genetic risk factor attributable to the highest prevalence of CRC in Jiamusi people. Individuals with CYP24A1 gene polymorphism may have an increased barrier for vitamin D absorption, thus contributing to the risk of CRC development.

## Introduction

Colorectal cancer (CRC) is the third most common malignant tumor in the world and the second leading cause of cancer death [1]. The annual cost of cancer is 124.6 billion dollars in the United States, 89.2 billion Euros in the European Union, and more than 220 billion rmb in China [2–4]. CRC has emerged as a major public health concern in China, having had 306.1 thousand new cases and 164.8 thousand deaths reported by the Global Cancer Observatory in 2020 [5]. The occurrence and development of CRC are thought to be strongly associated with genetic and environmental factors.

Jiamusi, which is situated at the northernmost area of China, has the highest rate (28.28/100000) [6] of CRC in the Eastern region, and about one third (5.00/100000) higher than those in the Central (23.51/100000) and Western area (25.17/100000) [7]. To elucidate such differences in the prevalence rates observed, the distinct environmental factor in Jiamusi demands an analysis of its causal relationship and interaction with the genetic polymorphism in the population. Being in the northeast of China from 45°56’ to 48°28’ north latitude and 129°29’ to 135°5’ east longitude, Jiamusi is known as the "East Pole" of China where the sun first rises. The climate in this area is cold, the terrain is high in the southwest and low in the northeast where the cold northerly wind drives straight in. The average temperature is 3°C with about 225 days of freezing period annually. This chronic lack of sunshine for over 6 months per year with resultant low Vitamin D (VD) level may contribute to the increased risk of CRC. The blood VD level (16.18–17.19ng/ml) of those over 60 years old is lower than that (26.08–27.20 ng/ml) of people in the southern provinces, and the rate of VD deficiency (69.89%) is higher compared with the Guangzhou population (16.63%) [8]. Thus, such a climatic environmental factor coupled with low VD level and increased CRC prevalence in the Jiamusi population may be associated with a higher frequency of VD-related gene variation.

Several studies have implicated VD deficiency in a variety of tumors including CRC, and such CRC susceptibility has been associated with the CYP24A1 gene mutations, of which the mechanism is related to specific vitamin D metabolic disorders in the body. Moreover, CYP24A1 has been demonstrated to promote tumorigenesis through the WNT pathway [9]. However, the association between CRC susceptibility and CYP24A1 gene polymorphism in the Jiamusi population in China is currently unknown.

CYP24A1 is a member of the cytochrome P450 enzyme family and the key enzyme that catabolizes 1,25(OH)2D3 which will be degraded through 24-hydroxylation, which is the rate-limiting step in vitamin D3 metabolism [10, 11]. With the first identification of CYP24A1 in breast cancer as a candidate oncogene [12], an increased or decreased CYP24A1 expression has been identified distinctively in various cancers such as prostate, endometrial, and lung [13–15]. A study by Sun et al. [16] has demonstrated a higher level of CYP24A1 expression in CRC tissues than in adjacent normal colorectal tissues. Therefore, CYP24A1 may represent a candidate oncogene for CRC. This study aimed to identify the relationship between the CYP24A1 gene polymorphism and CRC in the Jiamusi population. The Clinical-pathological features associated with specific CYP24A1 gene polymorphisms were studied.

## Materials and methods

### Study population

Of those patients admitted to the Department of Anorectal Surgery at the First Affiliated Hospital of Jiamusi University from March 2017 to December 2019, 168 patients with confirmed CRC having undergone an operation were recruited in the experimental group and 206 were included as controls. The clinical diagnostic criteria in our study were determined by colonoscopy and pathology results, which were adopted from the National Comprehensive Cancer Network (NCCN, https://www.nccn.org/). Demographic data were collected during in-person interviews, included age, sex, and residential region. A total of 710 patients including those with confirmed benign ano-colorectal pathology (n = 206) and individuals of the East Asian population of the Thousand People Genome Database (n = 504) were selected in the control group. All study participants did not have a kinship with each other. Blood samples and clinical-pathological data of all study participants were collected. The study was approved by the First Affiliated Hospital of Jiamusi University and Beijing Hospital Ethics Committee, and written informed consent was obtained from all subjects.

### SNP selection and genotyping

A total of 3ml venous blood was collected from each participant to extract DNA, and all DNA samples and data were handled anonymously. Genomic DNA was extracted by TAKARA whole blood genomic DNA extraction kit (centrifugal column type, Catalog No. 9781, Baori Medical Biotechnology (Beijing) Co., Ltd.). Quantitative DNA was quantified at 260nm using an ultraviolet absorption and stored at -80°C.

The human CYP24A1 gene is located in chromosome 20(20q 13.2) region, composed of eleven introns and twelve exons. Using the National Center for Biotechnology Information (NCBI) database to obtain the target gene sequence, we sequenced the complete coding sequence (12 exons, including intron/exon boundaries). All primers (S5 Table in S1 File) were synthesized by the TIAN YI Beijing Branch of Biological Co., Ltd. A random 17 CRC patients were selected for sequencing and the sequencing results were compared with a database of 1,000 genomes. There was no significant difference among the groups (p> 0.05) (S1 Table in S1 File). Then, a further random sample was extracted (60 subjects, 3 of whom had incomplete phenotypes). The DNA fragments corresponding to the SNP sites in relatively concentrated positions were selected to expand the sample. Three SNP sites of rs6013905, rs2762939, and rs6068816 were selected for this study (these sites belonged to the same DNA fragment and the rs2762939 allele (C/G) P<0.2, and these SNPs had minor allele frequency (MAF) ≥ 5% in the Hap-Map CHB population (S2 Table in S1 File).

### Amplification of gene fragments

Polymerase chain reaction (PCR) was carried out by using the Bio-Rad CFX 96 real-time PCR system (Bio-Rad, USA). We established a PCR reaction system (20 μl): Taq PCR Master Mix (2X) 10ul; 10p mol/ul Forward primer 0.4ul; 10p mol/ul Reverse primer 0.4ul; Template DNA 2ul; ddH_2_O 7.6ul; The reaction conditions: pre-denaturation at 95°C for 3min, denaturation at 95°C for 30s, annealing temperature of 25s at Tm, elongation at 72°C for 30s, duplicate 36 cycles, terminal elongation at 72°C for 10 min.

### Statistical analysis

The allele and genotype frequencies were calculated directly. SPSS20.0 software was used for statistical analysis. Measurement data were expressed as mean±standard deviation ($\bar{\text{x}}\pm \text{sd}$). The odds ratio (OR) and 95% Confidence Interval (95%CI) represented the gene polymorphism sites and the susceptibility risk of colorectal cancer. Hardy-Weinberg equilibrium was analyzed by 2test. The cut-off value of significant difference was P≤0.05.

## Results

1. Through the genetic association analysis of 168 CRC cases and 710 controls, a significant association between CYP24A1 polymorphism carriers (rs6013905AX and rs2762939GX) and CRC (p≤0.05) (Tables 1 and 2) was identified. Compared with the control group, CRC patients carrying rs6013905 GA genotype (P = 0.04, OR = 1.79, 95%CI: 1.01–3.19) and AA genotype (P = 0.02, OR = 2.02, 95%CI 1.13–3.63) had a significantly increased incidence risk. Also, the frequency of the rs6013905 A allele was associated with an increased incidence risk of CRC (P = 0.03, OR = 1.32, 95%CI: 1.03–1.69). The rs6013905 polymorphism had a significant association with CRC in the dominant model (P = 0.02, OR = 1.89, 95%CI: 1.09–3.29). Compared with the control group, CRC patients who carried the rs2762939 GC genotype were significantly at a higher incidence risk (P = 5.56*10–3, OR = 1.63, 95%CI: 1.15–2.31). Carriers of rs2762939 (GX) genotype were also significantly associated with an increased risk of CRC (P = 0.02, OR = 1.52, 95%CI: 1.08–2.13) in the dominant model. For CRC in the Jiamusi population, the calculated population-attributable risks for the associated genetics were variable, of which CYP24A1 rs6013905AX was 18.67%, rs2762939GX was 13.12%, and the accumulated risk was 29.34%.

**Table 1:** Characteristics of the study population.

Variable	Case No. (%)	Control No. (%)	P-value
	168	206	
**Age (years)**			N.S.
≤60	63 (37.50)	97 (47.09)	
>60	105 (62.50)	109(52.91)	
**Mean ± SD**	62.99±10.91	59.42±14.99	
**Sex**			N.S.
Male	98 (58.33)	112 (54.36)	
Female	70 (41.67)	94 (45.63)	
**Location of the primary tumor**			
Colon	96 (57.14)		
Rectum	72 (42.85)		
**General classification of tumors**			
Ulcerative type	130 (77.38)		
Uplift type	34 (20.24)		
**Degree of differentiation**			
Low	153 (91.07)		
High	9 (5.35)		
**TNM**			
I–II	83(49.40)		
III	81(48.21)		

N.S. represent no significant.

**Table 2:** Comparative analysis of CYP24A1 polymorphisms between the CRC and control groups.

Genotype	Case(freq), N = 168	Control(freq), N = 710	*P*	OR (95%CI)
**rs6013905**				
GG	16(0.10)	118(0.17)	Ref.	
GA	81(0.48)	333(0.47)	0.04	1.79(1.01–3.19)
AA	71(0.42)	259(0.36)	0.02	2.02(1.13–3.63)
Dominant model (AX)	152(0.90)	592(0.83)	0.02	1.89(1.09–3.29)
Recessive model (AA)	71(0.42)	259(0.36)	N.S.	
G	113(0.34)	569(0.40)	Ref.	
A	223(0.66)	851(0.60)	0.03	1.32(1.03–1.69)
**rs2762939**				
CC	77(0.46)	399(0.56)	Ref.	
CG	83(0.49)	264(0.37)	5.56*10^−3^	1.63(1.15–2.31)
GG	8(0.05)	47(0.07)	N.S.	
Dominant model (GX)	91(0.54)	311(0.44)	0.02	1.52(1.08–2.13)
Recessive model (GG)	8(0.05)	47(0.07)	N.S.	
C	237(0.71)	1062(0.75)	Ref.	
G	99(0.29)	358(0.25)	N.S.	
**rs6068816**				
GG	69(0.41)	261(0.37)	Ref.	
GA	81(0.48)	341(0.48)	N.S.	
AA	18(0.11)	108(0.15)	N.S.	
Dominant model (AX)	99(0.59)	449(0.63)	N.S.	
Recessive model (AA)	18(0.11)	108(0.15)	N.S.	
G	219(0.65)	863(0.61)	Ref.	
A	117(0.35)	557(0.39)	N.S.	

**Fig 1:**
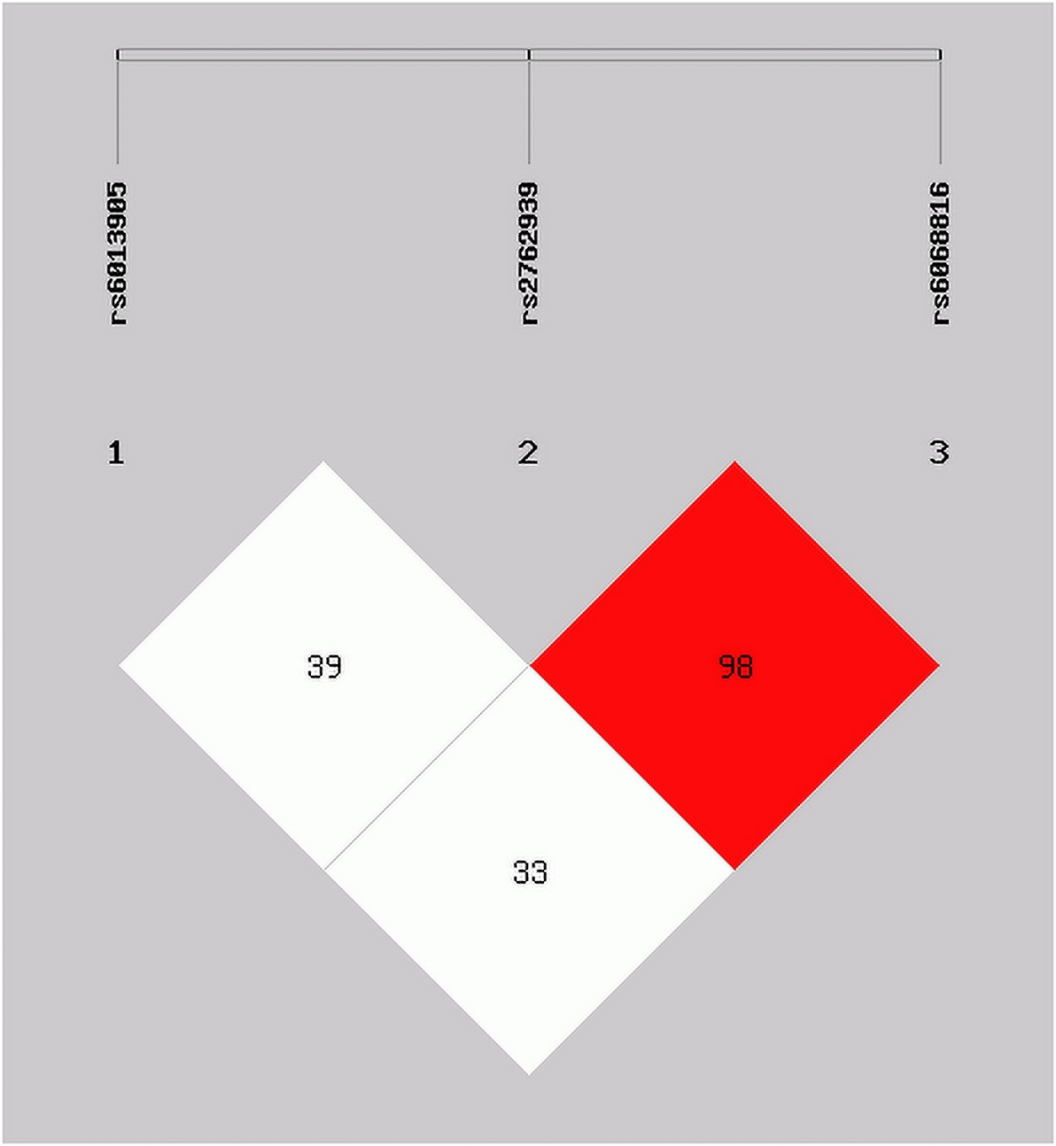
Linkage disequilibrium analysis of CYP24A1 polymorphisms (rs6013905, rs2762939 and rs6068816).

2. The linkage disequilibrium analysis detected that there was a block with significance (D’ = 0.98), formed between rs2762939 and rs6068816 on CYP24A1 (Fig 1), but no markedly linkage disequilibrium between rs6013905 and rs2762939 or rs6068816 was demonstrated. There were seven haplotypes CYP24A1 polymorphism altogether, compared with the reference haplotype ACA of lowest risk with CRC. We observed that the ACG (P = 2.10×10–5, OR:1.74, 95%CI: 1.35–2.26), AGG (P = 3.31×10–31, OR: 5.98, 95%CI: 4.30–8.31), and GCA (P = 1.97×10–26, OR: 4.70, 95%CI: 3.47–6.37) increased the risk of CRC. The haplotypes GCG (P = 1.99×10–7, OR:0.20, 95%CI: 0.10–0.39) and GGG (P = 7.32×10–15, OR: 0.08, 95%CI: 0.04–0.18) reduced the risk of CRC (Table 3).

**Table 3:** Haplotype analysis of CYP24A1 polymorphism between the CRC and control groups: rs6013905, rs2762939, rs6068816.

	Case (freq)	Control (freq)	Chi^2^	*P*-value	OR (95%CI)
ACA	17(0.05)	442(0.31)	95.29	1.75*10–22	0.12(0.07–0.19)
ACG	114(0.34)	326(0.23)	18.12	2.10*10–05	1.74(1.35–2.26)
AGG	89(0.27)	82(0.06)	135.16	3.31*10–31	5.98(4.30–8.31)
GCA	97(0.29)	114(0.08)	113.32	1.97*10–26	4.703(3.47–6.37)
GCG	9(0.03)	180(0.13)	27.08	1.99*10–07	0.201(0.10–0.39)
GGG	7(0.02)	275(0.19)	60.6	7.32*10–15	0.083(0.04–0.18)

**Fig 2:**
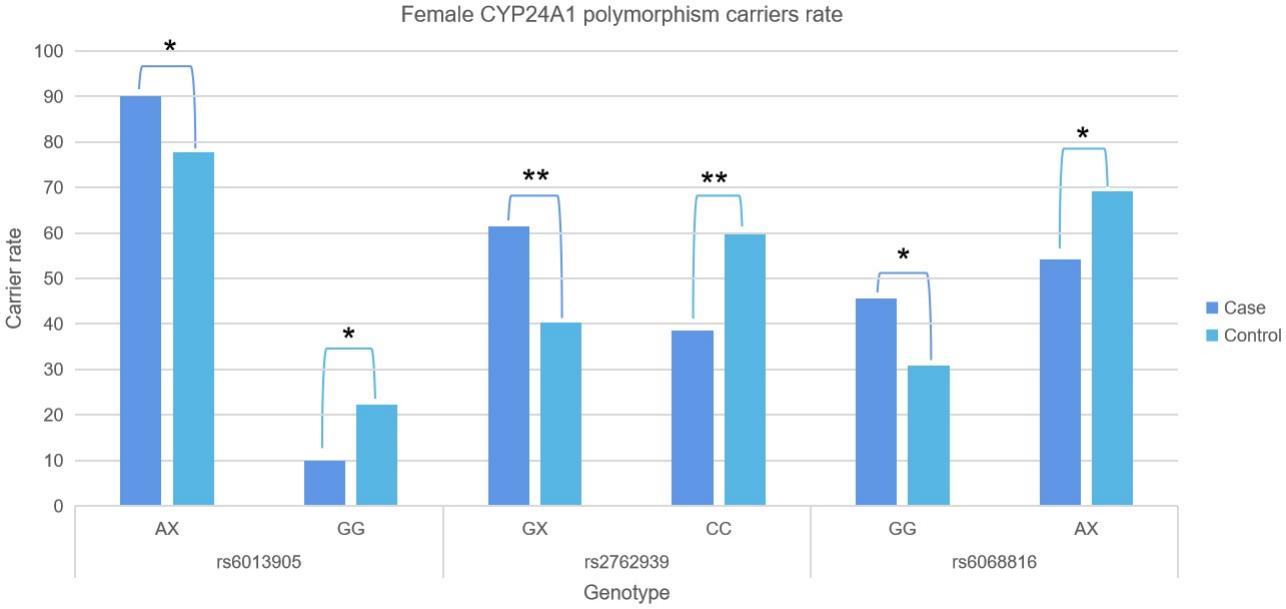
Comparison of CYP24A1 genetic polymorphism carrier frequencies between the CRC and control groups. * denoted p≤0.05; ** p<0.01.

3. Through gender stratification, our analysis ascertained that females with CYP24A1 polymorphism rs6013905 AX (P = 0.04, OR = 2.59, 95%CI: 1.03–6.49), rs2762939 GX (P = 0.01, OR = 2.35, 95%CI: 1.25–4.42), and rs6068816 GG (P = 0.05, OR = 1.89, 95%CI: 0.99–3.59) carriers were significantly associated with CRC (Fig 2, S3 Table in S1 File).

4. Through the case-only study of genotype-phenotype analysis, CYP24A1 polymorphism rs6068816 AX was significantly associated with colon cancer (P = 0.03, OR = 2.08, 95%CI: 1.10–3.96), not rectal cancer. Furthermore, CYP24A1 polymorphism rs6068816 AX carriers were significantly associated with the intestinal ulcer in patients with CRC (P = 0.04, OR = 2.24, 95%CI: 1.05–4.77). In addition, the other two polymorphisms rs6013905 GG (P = 0.05, OR = 4.00, 95%CI: 0.92–17.48) and rs2762939 GX (P = 0.03, OR = 4.87, 95%CI: 1.00–23.69) carriers were significantly associated with poorly differentiated CRC (Table 4).

**Table 4:** Case-only analysis of genotypes and phenotypes in the CRC.

Subgroups	rs6013905				rs2762939				rs6068816			
	GG/AX		*P*	OR (95%CI)	GX /CC		*P*	OR (95%CI)	AX /GG		*P*	OR (95%CI)
Location of the primary tumor			0.44				0.59				0.03	2.08 (1.10–3.96)
Colon	10 (0.50)	58 (0.41)			34 (0.40)	34 (0.44)			44 (0.50)	24 (0.32)		
Rectum	10 (0.50)	84 (0.59)			51 (0.60)	43 (0.56)			44 (0.50)	50 (0.68)		
General classification of tumors			0.75				0.24				0.04	2.24 (1.05–4.77)
ulcerative type	15 (0.75)	111 (0.78)			63 (0.74)	63 (0.82)			74 (0.84)	52 (0.70)		
Uplift type	5 (0.25)	31 (0.22)			22 (0.26)	14 (0.18)			14 (0.16)	22 (0.30)		
Degree of differentiation			0.05	4.00 (0.92–17.48)			0.03	4.87 (1.00–23.69)			0.15	
Low	3 (0.15)	6 (0.04)			84 (0.98)	69 (0.90)			81 (0.92)	72 (0.97)		
High	17 (0.85)	136 (0.96)			2 (0.02)	8 (0.10)			7 (0.08)	2 (0.03)		

## Discussion

The frequency of the gene polymorphism of CYP24A1 associated with colon cancer is relatively constant among the various population in the world (Europe and China). However, the prevalence of CRC varies greatly in these regions. Our analysis suggested that the environmental factor plays an important “tuner” for CRC susceptible risk in the population, for example, the length of local annual sunshine time, and the average level of vitamin D. The probability of a direct association between the differences in the length of annual sunshine time, vitamin D levels and CYP24A1-associated-CRC demands a further study. Given that individuals living in the areas with low sunlight would have a reduced level of vitamin D synthesized in their body (S2 Fig in S1 File), the carrier of the vitamin D metabolism-related gene CYP24A1 mutation may be associated with a further reduced level or loss of the physiological function of vitamin D, which may then increase the susceptibility to CRC or trigger the CRC carcinogenesis (Table 4). As a result of short sunshine exposure time and skin covered under thick clothes, individuals living in the high latitude area of Jiamusi have a reduction of VD synthesis in their skin and therefore prone to VD deficiency. In addition, the synthesis of VD in the skin can be affected by many factors, such as cloud thickness, air pollution, long winter season, and staying indoors for a prolonged period of time. Furthermore, not only the amount of sunlight but the intensity of ultraviolet rays may affect the VD synthesis. When the ultraviolet rays irradiate the skin, the heptadehydrocholesterol under the skin is converted into 1,25–2 hydroxy vitamin D3. The 1,25(OH)2D3(calcitriol), as a hormone form of vitamin D, has several effects on cell homeostasis, including proliferation, apoptosis, differentiation, and inflammation [17]. Among the many genes induced by calcitriol, CYP24A1 (also known as 24-hydroxylase) is particularly of importance. It encodes the enzyme that catalyzes the degradation of both 1,25(OH) 2D3 (calcitriol) and 25(OH)D. The enzyme is key in breaking down the 1,25(OH)2D3 into the less active 25-d3 [18]. A study has shown a higher level of expression of CYP24A1 mRNA and protein in CRC tissue than that in non-cancer tissue [16], while another study has confirmed an absence or reduced CYP24A1 expression level normal colonic mucosa [19]. Based on these studies, we hypothesized that CYP24A1 may play a crucial role in the pathogenesis of cancer.

At present, there are limited studies examining the relationship between rs6013905 and CRC. In our study, rs6013905 demonstrated a statistically significant association with the risk of CRC when both genotypes and allele frequencies were considered. Compared with G in colon cancer patients, the minimum OR of rs6013905 A was 1.319 (P = 0.03). Our results indicated that the A allele is a strong risk factor. Changes in rs6013905 would not affect the function or structure of proteins encoded by CYP24A1 but may affect its intron splice.

The rs2762939 has previously been studied on prostate cancer [20], non-small cell lung cancer [21], coronary atherosclerosis [22], and non-Hodgkin’s lymphoma [23]. Our study has shown that carriers of rs2762939 GX genotype (P = 0.02, OR = 1.52, 95%CI: 1.08–2.13) and GC allele (P = 5.56*10–3, OR = 1.63, 95% CI: 1.15–2.31) were associated with a significantly increased risk of CRC. Interestingly, females rs2762939 (GX) carriers (P = 0.01, OR = 2.35, 95%CI: 1.25–4.42) had a higher incidence risk of CRC when compared with those in the control group. For rs2762939, the C allele and G allele of rs2762939 are a co-manifest allele in CRC. The study by Varakantham et al. [24] has found a negative correlation of rs2762939 CC variant with essential hypertension in women in the Indian population, which also suggests that rs2762939 has gender propensity but further research is needed.

The association of rs6068816 with cancers has been variably reported. Chen et al. [25] has found that rs6068816 T is a strong risk factor for colon cancer in the Chinese population, On the other hand, Wu et al. [26] have reported that CYP24A1 rs6068816 is significantly related to the decreased risk of non-small cell lung cancer (NSCLC) development among the Chinese. Consistent with this, Li et al. [27] have also found that rs2762934 and rs6068816 in CYP24A1 are protective factors to lung cancer (LC) in males and small cell lung cancer (SCLC) respectively. In our study, we have demonstrated that the frequency of rs6068816 GG genotypes in women was associated with a significantly increased risk of CRC. Given that the amino acid sequence of CYP24A1 is not affected by rs6068816 base variation, the SNP located in the silencer or enhancer of the splicing region can affect the phenotype of biological activity by affecting the mRNA splicing efficiency.

Previous studies have shown an abnormally increased level of CYP24A1 expression in mRNA of CRC tissues [18], and the relative expression was significantly higher when compared with normal adjacent tissues [28]. Our study has further shown a significant correlation of CYP24A1 expression with several clinical features, including the lesion site, general type, and histologic type of CRC, which confirms a pro-oncogenic effect of CYP24A1.

There were some limitations to our present study. The occurrence and development of a tumor is a very complicated process, which requires the interaction of internal and external factors. Our research provided evidence on the relationship between gene polymorphism and genetic susceptibility of CRC but the specific pathogenesis and the correlation with the prognosis of the tumor would warrant further study. In addition, the sample size was relatively small, and all cases were from a single institution only. More corroborative research including a wider study population and regions would be required to validate our findings.

In conclusion, this study stipulates an association of CYP24A1 genes with CRC, and it may be that CYP24A1 is involved in CRC carcinogenesis. This would shed light on the public health significance of CRC prevention in a specific population, such as through sunlight exposure or supplementing VD to individuals in a low sunshine exposure area, or developing an early effective intervention for identifying CYP24A1 variants carriers.

## Supporting information

S1 Checklist *PLOS ONE* clinical studies checklist. (DOCX)

S2 Checklist STROBE statement—Checklist of items that should be included in reports of observational studies. (DOCX)

S1 File (DOCX)

## References

[1] Keum N, Giovannucci E. Global burden of colorectal cancer: emerging trends, risk factors and prevention strategies. Nat Rev Gastroenterol Hepatol. 2019;16(12):713–732. doi: 10.1038/s41575-019-0189-8 31455888

[2] Jönsson B, Hofmarcher T, Lindgren P, Wilking N. The cost and burden of cancer in the European Union 1995–2014. Eur J Cancer. 2016;66:162–170. doi: 10.1016/j.ejca.2016.06.022 27589247

[3] Mariotto AB, Yabroff KR, Shao Y, Feuer EJ, Brown ML. Projections of the cost of cancer care in the United States: 2010–2020. J Natl Cancer Inst. 2011;103(2):117–128. doi: 10.1093/jnci/djq495 21228314 PMC3107566

[4] China spends more than 220 billion yuan a year on cancer care, according to the National Cancer Center. Shanghai pharma. 2019;40:03.

[5] World Health Organization. Global Cancer Observatory. International Agency for Research on Cancer. 2020. https://gco.iarc.fr/today/data/factsheets/populations/160-china-fact-sheets.pdf

[6] Sun H, Chen W, Zhang M, Song B. Analysis of cancer incidence and mortality in Heilongjiang cancer registries, 2014. Chin J Cancer Prev Treat. 2018;25(20):1407–1412.

[7] Chen WQ, Sun KX, Zheng RS. Report of Cancer Incidence and Mortality in Different Areas of China, 2014. China Cancer. 2018;27(01):1–14. 10.3760/cma.j.issn.0253-3766.2018.01.002 29365411

[8] Xiao L, Xu Y, Cheng Y, Tao X, Dai S, Yang WH. Investigation of serum 25-hydroxyvitamin D level in 10696 elderly people over 60 years old. Int J Lab Med. 2020;41(05):578–582.

[9] Sun H, Jiang C, Cong L, Wu N, Wang X, Hao M, et al. CYP24A1 Inhibition Facilitates the Antiproliferative Effect of 1,25(OH)2D3 Through Downregulation of the WNT/β-Catenin Pathway and Methylation-Mediated Regulation of CYP24A1 in Colorectal Cancer Cells. DNA Cell Biol. 2018;37(9):742–749. doi: 10.1089/dna.2017.4058 30052060

[10] Jones G, Prosser DE, Kaufmann M. 25-Hydroxyvitamin D-24-hydroxylase (CYP24A1): Its important role in the degradation of vitamin D. Arch Biochem Biophy. 2012;523(1):9–18. doi: 10.1016/j.abb.2011.11.003 22100522

[11] Deeb KK, Trump DL, Johnson CS. Vitamin D signaling pathways in cancer: potential for anticancer therapeutics. Nat Rev Cancer. 2007;7(9):684–700. doi: 10.1038/nrc2196 17721433

[12] Albertson DG, Ylstra B, Segraves R, Collins C, Dairkee SH, Kowbel D, et al. Quantitative mapping of amplicon structure by array CGH identifies CYP24 as a candidate oncogene. Nat Genet. 2000;25(2):144–146. doi: 10.1038/75985 10835626

[13] Bokhari AA, Lee LR, Raboteau D, Turbov J, Rodriguez IV, Pike JW, et al. Progesterone potentiates the growth inhibitory effects of calcitriol in endometrial cancer via suppression of CYP24A1. Oncotarget. 2016;7(47):77576–77590. doi: 10.18632/oncotarget.12725 27769055 PMC5363606

[14] Chen G, Kim SH, King AN, Zhao L, Simpson RU, Christensen PJ, et al. CYP24A1 is an independent prognostic marker of survival in patients with lung adenocarcinoma. Clin Cancer Res.2011;17(4):817–826. doi: 10.1158/1078-0432.CCR-10-1789 21169243 PMC3058389

[15] Whitlatch LW, Young MV, Schwartz GG, Flanagan JN, Burnstein KL, Lokeshwar BL, et al. 25-Hydroxyvitamin D-1α-hydroxylase activity is diminished in human prostate cancer cells and is enhanced by gene transfer. J Steroid Biochem Mol Biol. 2002;81(2):135–140. doi: 10.1016/s0960-0760(02)00053-5 12137802

[16] Sun H, Wang C, Hao M, Sun R, Wang Y, Liu T, et al. CYP24A1 is a potential biomarker for the progression and prognosis of human colorectal cancer. Hum Pathol. 2016;50:101–108. doi: 10.1016/j.humpath.2015.11.008 26997443

[17] Feldman D, Krishnan AV, Swami S, Giovannucci E, Feldman BJ. The role of vitamin D in reducing cancer risk and progression. Nat Rev Cancer. 2014;14(5):342–357. doi: 10.1038/nrc3691 24705652

[18] Muindi JR, Adjei AA, Wu ZR, Olson I, Huang H, Groman A, et al. Serum vitamin D metabolites in colorectal cancer patients receiving cholecalciferol supplementation: correlation with polymorphisms in the vitamin D genes. Horm Cancer. 2013;4(4):242–250. doi: 10.1007/s12672-013-0139-9 23456391 PMC3689467

[19] Horváth HC, Lakatos P, Kósa JP, Bácsi K, Borka K, Bises G, et al. The candidate oncogene CYP24A1: A potential biomarker for colorectal tumorigenesis. J Histochem Cytochem.2010;58(3):277–285. doi: 10.1369/jhc.2009.954339 19901270 PMC2825493

[20] Holt SK, Kwon EM, Koopmeiners JS, Stepic J, Pejovic M, Colic S, et al. Vitamin D pathway gene variants and prostate cancer prognosis. Prostate. 2010;70(13):1448–1460. doi: 10.1002/pros.21180 20687218 PMC2927712

[21] Ramnath N, Daignault-Newton S, Dy GK, Muindi JR, Adjei A, Elingrod VL, et al. A phase I/II pharmacokinetic and pharmacogenomic study of calcitriol in combination with cisplatin and docetaxel in advanced non-small-cell lung cancer. Cancer Chemother Pharmacol. 2013;71(5):1173–1182. doi: 10.1007/s00280-013-2109-x 23435876 PMC3637851

[22] Shen H, Bielak LF, Ferguson JF, Streeten EA, Yerges-Armstrong LM, Liu J, et al. Association of the vitamin D metabolism gene CYP24A1 with coronary artery calcification. Arterioscl Throm Vas. 2010;30(12):2648–2654. doi: 10.1161/ATVBAHA.110.211805 20847308 PMC2988112

[23] Kelly JL, Drake MT, Fredericksen ZS, Asmann YW, Liebow M, Shanafelt TD, et al. Early life sun exposure, vitamin D-related gene variants, and risk of non-Hodgkin lymphoma. Cancer Causes Control. 2012;23(7):1017–1029. doi: 10.1007/s10552-012-9967-0 22544453 PMC3589750

[24] Varakantham V, Ale K, Sailoo AKK, Nagalla B, Bharatraj DK. Sex-specific role of CYP24A1 rs2762939 in the risk of essential hypertension based on the serum vitamin D and total renin concentrations. Genomics. 2020;112(1):764–768. doi: 10.1016/j.ygeno.2019.05.013 31102703

[25] Chen XQ, Mao JY, Li WB, Li J, Yang H, Qian JM, et al. Association between CYP24A1 polymorphisms and the risk of colonic polyps and colon cancer in a Chinese population. World J Gastroenterol. 2017;23(28):5179–5186. doi: 10.3748/wjg.v23.i28.5179 28811712 PMC5537184

[26] Wu X, Cheng J, Yang K. Vitamin D-Related Gene Polymorphisms, Plasma 25-Hydroxy-Vitamin D, Cigarette Smoke and Non-Small Cell Lung Cancer (NSCLC) Risk. Int J Mol Sci. 2016;17(10):1597. doi: 10.3390/ijms17101597 27669215 PMC5085630

[27] Li M, Li A, He R, Dang W, Liu X, Yang T, et al. Gene polymorphism of cytochrome P450 significantly affects lung cancer susceptibility. Cancer Med. 2019;8(10):4892–4905. doi: 10.1002/cam4.2367 31264381 PMC6712450

[28] Wang L, Liu L. The Expression of CDX2 and CYP24A1 in Colorectal Cancer. *Med Inform*. 2018;31(7):72.

