# Supplementary material for: Association of CYP24A1 gene polymorphism with colorectal cancer in the Jiamusi population
Source: PLoS One. 2021 Jun 30;16(6):e0253474. doi: 10.1371/journal.pone.0253474 (PMC8244863; doi:10.1371/journal.pone.0253474)
Supplement: S2 Checklist — (DOCX) [file pone.0253474.s002.docx]

STROBE Statement—checklist of items that should be included in reports of observational studies

|  | Item No. | Recommendation | Page  No. | Relevant text from manuscript |
| --- | --- | --- | --- | --- |
| **Title and abstract** | 1 | (*a*) Indicate the study’s design with a commonly used term in the title or the abstract | 1 | Association of CYP24A1 gene polymorphism with colorectal cancer in the Jiamusi population |
|  |  | (*b*) Provide in the abstract an informative and balanced summary of what was done and what was found | 2 | Given the long cold seasons with people wearing more clothes and reduced UV exposure, we aimed to study the association between vitamin D receptor CYP24A1 gene polymorphism and CRC susceptibility. vitamin D receptor CYP24A1 gene polymorphism may be a genetic risk factor attributable to the highest prevalence of CRC in Jiamusi people. |
| Introduction | | | |  |
| Background/rationale | 2 | Explain the scientific background and rationale for the investigation being reported | 3 and 4 | The occurrence and development of CRC are thought to be strongly associated with genetic and environmental factors. Jiamusi, which is situated at the northernmost area of China, has the highest rate (28.28/100000) of CRC in the Eastern region. Thus, such a climatic environmental factor coupled with low VD level and increased CRC prevalence in the Jiamusi population may be associated with a higher frequency of VD-related gene variation. Several studies have implicated VD deficiency in a variety of tumors including CRC, and such CRC susceptibility has been associated with the VD receptor CYP24A1 gene mutations, of which the mechanism is related to specific metabolic disorders as VD receptor varies in the body. Moreover, CYP24A1 has been demonstrated to promote tumorigenesis through the WNT pathway. |
| Objectives | 3 | State specific objectives, including any prespecified hypotheses | 4 | This study aimed to identify the relationship between the CYP24A1 gene polymorphism and CRC in the Jiamusi population. The Clinical-pathological features associated with specific CYP24A1 gene polymorphisms were studied. |
| Methods | | | |  |
| Study design | 4 | Present key elements of study design early in the paper | 4 and 5 | patients with confirmed CRC and controls, CYP24A1 gene, Clinical-pathological phenotype |
| Setting | 5 | Describe the setting, locations, and relevant dates, including periods of recruitment, exposure, follow-up, and data collection | 4 | Under the entry of “Study population” |
| Participants | 6 | (*a*) *Cohort study*—Give the eligibility criteria, and the sources and methods of selection of participants. Describe methods of follow-up  *Case-control study*—Give the eligibility criteria, and the sources and methods of case ascertainment and control selection. Give the rationale for the choice of cases and controls  *Cross-sectional study*—Give the eligibility criteria, and the sources and methods of selection of participants | 4 | 168 patients with confirmed CRC having undergone an operation were recruited in the experimental group |
|  |  | (*b*) *Cohort study*—For matched studies, give matching criteria and number of exposed and unexposed  *Case-control study*—For matched studies, give matching criteria and the number of controls per case | 4 | 168 patients with confirmed CRC having undergone an operation were recruited in the experimental group. The clinical diagnostic criteria in our study is from National Comprehensive Cancer Network (NCCN, https://www.nccn.org/). |
| Variables | 7 | Clearly define all outcomes, exposures, predictors, potential confounders, and effect modifiers. Give diagnostic criteria, if applicable | 4 | 168 patients with confirmed CRC having undergone an operation were recruited in the experimental group. The clinical diagnostic criteria in our study is from National Comprehensive Cancer Network (NCCN, https://www.nccn.org/). |
| Data sources/ measurement | 8* | For each variable of interest, give sources of data and details of methods of assessment (measurement). Describe comparability of assessment methods if there is more than one group | 4 | Colonoscopy and pathology results |
| Bias | 9 | Describe any efforts to address potential sources of bias | 5 | We collected blood samples and clinical-pathological features of all study participants. |
| Study size | 10 | Explain how the study size was arrived at | 4 | Of the 168 cases and 206 controls admitted to the Department of Anorectal Surgery at the First Affiliated Hospital of Jiamusi University from March 2017 to December 2019, 168 patients with confirmed CRC having undergone an operation were recruited in the experimental group |

Continued on next page

| Quantitative variables | 11 | Explain how quantitative variables were handled in the analyses. If applicable, describe which groupings were chosen and why | 4 | Colonoscopy and pathology results |
| --- | --- | --- | --- | --- |
| Statistical methods | 12 | (*a*) Describe all statistical methods, including those used to control for confounding |  |  |
|  |  | (*b*) Describe any methods used to examine subgroups and interactions |  |  |
|  |  | (*c*) Explain how missing data were addressed |  |  |
|  |  | (*d*) *Cohort study*—If applicable, explain how loss to follow-up was addressed  *Case-control study*—If applicable, explain how matching of cases and controls was addressed  *Cross-sectional study*—If applicable, describe analytical methods taking account of sampling strategy | 6 | The allele and genotype frequencies were calculated directly. SPSS20.0 software was used for statistical analysis. Measurement data were expressed as mean±standard deviation (`x±sd). The odds ratio (OR) and 95% Confidence Interval (95%CI) represented the gene polymorphism sites and the susceptibility risk of colorectal cancer. Hardy-Weinberg equilibrium was analyzed by 2test. The cut-off value of significant difference was P <0.05. |
|  |  | (*e*) Describe any sensitivity analyses |  |  |
| Results | | | | |
| Participants | 13* | (a) Report numbers of individuals at each stage of study—eg numbers potentially eligible, examined for eligibility, confirmed eligible, included in the study, completing follow-up, and analysed | 6 | 168 CRC cases and 710 controls |
|  |  | (b) Give reasons for non-participation at each stage |  |  |
|  |  | (c) Consider use of a flow diagram |  |  |
| Descriptive data | 14* | (a) Give characteristics of study participants (eg demographic, clinical, social) and information on exposures and potential confounders | 4 | the 168 cases and 206 controls;  a total of 710 patients including those with confirmed benign ano-colorectal pathology (n = 206) and individuals of the East Asian population of the Thousand People Genome Database (n = 504) were selected in the control group. |
|  |  | (b) Indicate number of participants with missing data for each variable of interest |  |  |
|  |  | (c) *Cohort study*—Summarise follow-up time (eg, average and total amount) |  |  |
| Outcome data | 15* | *Cohort study*—Report numbers of outcome events or summary measures over time |  |  |
|  |  | *Case-control study—*Report numbers in each exposure category, or summary measures of exposure | 4 | the 168 cases and 206 controls;  a total of 710 patients including those with confirmed benign ano-colorectal pathology (n = 206) and individuals of the East Asian population of the Thousand People Genome Database (n = 504) were selected in the control group. |
|  |  | *Cross-sectional study—*Report numbers of outcome events or summary measures |  |  |
| Main results | 16 | (*a*) Give unadjusted estimates and, if applicable, confounder-adjusted estimates and their precision (eg, 95% confidence interval). Make clear which confounders were adjusted for and why they were included | 6 | Compared with the control group, CRC patients carrying rs6013905 GA genotype (P = 0.044, OR = 1.794, 95%CI: 1.009-3.191) and AA genotype (P= 0.017, OR = 2.022, 95%CI 1.127-3.627) had a significantly increased incidence risk. |
|  |  | (*b*) Report category boundaries when continuous variables were categorized |  |  |
|  |  | (*c*) If relevant, consider translating estimates of relative risk into absolute risk for a meaningful time period |  |  |

Continued on next page

| Other analyses | 17 | Report other analyses done—eg analyses of subgroups and interactions, and sensitivity analyses | 7 | Through gender stratification, our analysis ascertained that females with CYP24A1 polymorphism rs6013905 AX (P=0.036, OR=2.589, 95%CI: 1.032-6.493), rs2762939 GX (P=0.008, OR=2.347, 95%CI: 1.246-4.422), and rs6068816 GG (P=0.05, OR=1.887, 95%CI: 0.99-3.59) carriers were significantly associated with CRC |
| --- | --- | --- | --- | --- |
| Discussion | | | | |
| Key results | 18 | Summarise key results with reference to study objectives | 9 | Our study has shown that carriers of rs2762939 GX genotype (P=0.015, OR=1.516, 95%CI: 1.082-2.125) and GC allele (P=5.558*10-3，OR =1.629, 95% CI: 1.152-2.305) were associated with a significantly increased risk of CRC. Interestingly, females rs2762939 (GX) carriers (P =0.008, OR=2.347, 95%CI: 1.246-4.422) had a higher incidence risk of CRC when compared with those in the control group. |
| Limitations | 19 | Discuss limitations of the study, taking into account sources of potential bias or imprecision. Discuss both direction and magnitude of any potential bias | 10 | The occurrence and development of a tumor is a very complicated process, which requires the interaction of internal and external factors. Our research provided the evidence on the relationship between gene polymorphism and genetic susceptibility of CRC but the specific pathogenesis and the correlation with the prognosis of the tumor would warrant further study. In addition, the sample size was relatively small, and all cases were from a single institution only. More corroborative research including a wider study population and regions would be required to validate our findings. |
| Interpretation | 20 | Give a cautious overall interpretation of results considering objectives, limitations, multiplicity of analyses, results from similar studies, and other relevant evidence | 10 | In our study, we have demonstrated that the frequency of rs6068816 GG genotypes in women was associated with a significantly increased risk of CRC. Given that the amino acid sequence of CYP24A1 is not affected by rs6068816 base variation, the SNP located in the silencer or enhancer of the splicing region can affect the phenotype of biological activity by affecting the mRNA splicing efficiency. |
| Generalisability | 21 | Discuss the generalisability (external validity) of the study results | 10 | Our study has further shown a significant correlation of CYP24A1 expression with several clinical features, including the lesion site, general type, and histologic type of CRC, which confirms a pro-oncogenic effect of CYP24A1 |
| Other information | |  | | |
| Funding | 22 | Give the source of funding and the role of the funders for the present study and, if applicable, for the original study on which the present article is based | 11 | This work is supported by grants from by the Natural Science Foundation of China (9184910151,81849132,81400790,81460203,3176029,81571385,91849132,81872096); National Key Research and Development Program of China (2018YFC2000400); Guangxi Natural Science Foundation (2014GXNSFDA118028, 2018GXNSFAA138156, guike0991198); Self-funded Scientific Research Project of the health and family planning commission of Guangxi Zhuang Autonomous Region (Z20170162); Beijing Hospital Nova Project (BJ-2018-139); Non-profit Central Research Institute Fund of Chinese Academy of Medical Sciences (2018RC330003). |

*Give information separately for cases and controls in case-control studies and, if applicable, for exposed and unexposed groups in cohort and cross-sectional studies.

**Note:** An Explanation and Elaboration article discusses each checklist item and gives methodological background and published examples of transparent reporting. The STROBE checklist is best used in conjunction with this article (freely available on the Web sites of PLoS Medicine at http://www.plosmedicine.org/, Annals of Internal Medicine at http://www.annals.org/, and Epidemiology at http://www.epidem.com/). Information on the STROBE Initiative is available at www.strobe-statement.org.
