# Supplementary material for: Association of CYP24A1 gene polymorphism with colorectal cancer in the Jiamusi population
Source: PLoS One. 2021 Jun 30;16(6):e0253474. doi: 10.1371/journal.pone.0253474 (PMC8244863; doi:10.1371/journal.pone.0253474)
Supplement: S1 File — (DOCX) [file pone.0253474.s003.docx]

**S1 File**

**Association of CYP24A1 gene polymorphism with colorectal cancer in the Jiamusi population**

Lin Chai^1,2^, Jian Ni^1#^, Xiaolin Ni^2,3^, Nan Zhang^2^, Zhaoping Wang^2^, Yang Liu^1#^, Zhiwu Ji^1#^, Xingwang Zhao^1#^, Xiaowen Zhu^1#^, Bin Zhao^1#^, Guorong Xin^1#^, Yu Wang^1#^, Fan Yang^1#^, Liang Sun^2^, Xiaoquan Zhu^2^, Wenhua Bao^1#^, Xiaofang Shui^1^, Fengling Wang^1^, Fujun Chen^1*^, Ze Yang^2,3*^

*^1^ The First Affiliated Hospital of Jiamusi University, Heilongjiang, P.R.China.*

*^2^ The Key Laboratory of Geriatrics，Beijing Institute of Geriatrics, Beijing Hospital, National Center of Gerontology, National Health Commission; Institute of Geriatric Medicine, Chinese Academy of Medical Sciences, Beijing, P.R.China.*

*^3^ Graduate School of Chinese Academy of Medical Science and Peking Union Medical College, Beijing, P.R.China.*

*Corresponding author:

Email: [yang_ze@sina.com](mailto:yang_ze@sina.com) (ZY); [gck8801079@163.com](mailto:gck8801079@163.com) (FC)

# These authors contributed equally to this work.

**Supporting figure legends**

**S1 Fig. The Annual Average Temperature in Jiamusi.**

**S2 Fig. The Prevalence of Colorectal Cancer and Annual Sunshine Hours in Different Regions of China.**

**Supporting table legends**

**S1 Table. Association between CYP24A1 Single Nucleic Acid Gene Polymorphism and the Risk of Colorectal Cancer.**

**S2 Table. Association between CYP24A1 Single Nucleic Acid Gene Polymorphism and the Risk of Colorectal Cancer.**

**S3 Table. Stratified Analysis of the Relationship between CYP24A1 Polymorphisms and CRC as well as its Risk Factors by Case-control.**

**S4 Table. Hardy - Weinberg of CYP24A1 SNP Locus Genotypes in the Control Group.**

**S5 Table. All Primer Sequences of CYP24A1.**


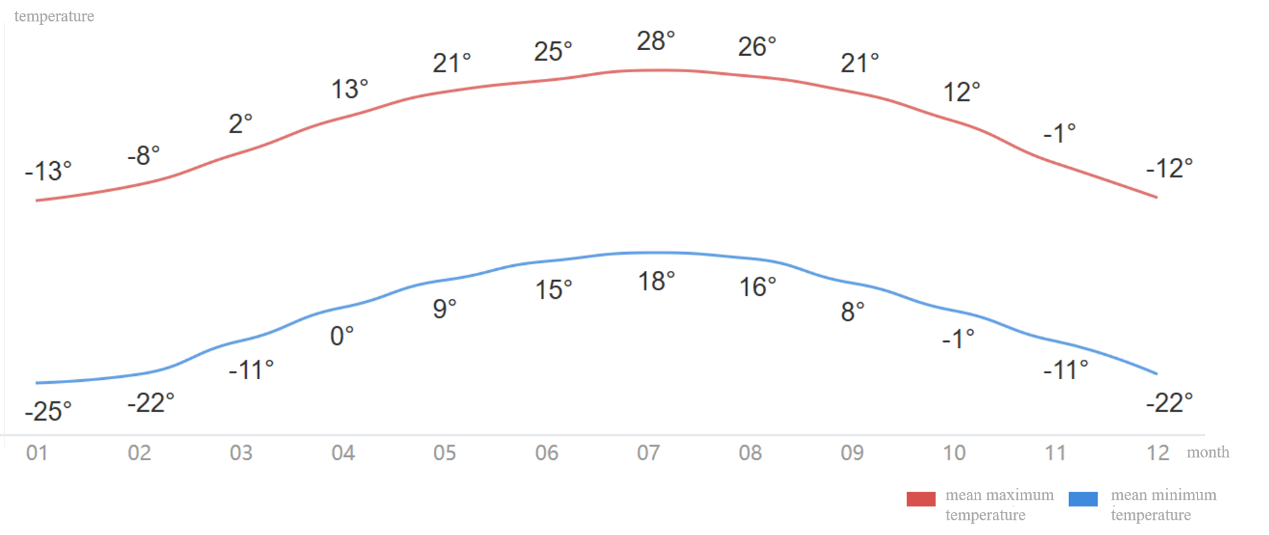


**S1 Fig. The Annual Average Temperature in Jiamusi.**


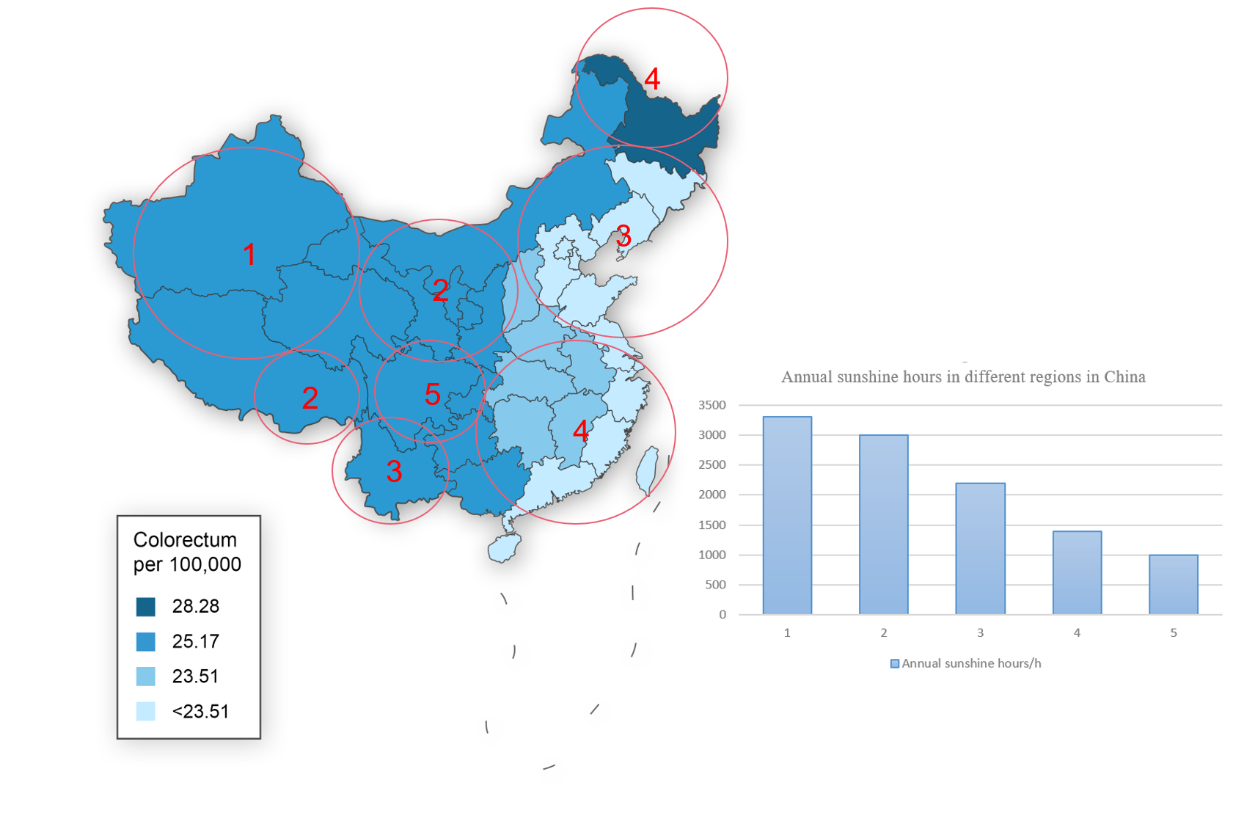


**S2 Fig. The Prevalence of Colorectal Cancer and Annual Sunshine Hours in Different Regions of China.**

**S1 Table.** Association between CYP24A1 single nucleic acid gene polymorphism and the risk of colorectal cancer

| Gene | SNP | Position | Change | Cases(n=17) | | Controls(n=504) | | *P* |
| --- | --- | --- | --- | --- | --- | --- | --- | --- |
|  |  |  |  | Minor allele | Major  allele | Minor  allele | Major  allele |  |
| CYP24A1 | rs2259735 | Chr20:52788314 | T/C | 24(0.706) | 10(0.294) | 659(0.654) | 349(0.346) | 0.529 |
| CYP24A1 | rs2245153 | Chr20:52786406 | T/C | 6(0.176) | 28(0.824) | 208(0.206) | 800(0.794) | 0.671 |
| CYP24A1 | rs4809958 | Chr20:52782438 | T/G | 8(0.235) | 26(0.765) | 395(0.392) | 613(0.608) | 0.065 |
| CYP24A1 | rs6013905 | Chr20:52781264 | A/G | 15(0.441) | 19(0.559) | 410(0.407) | 598(0.593) | 0.687 |
| CYP24A1 | rs2762939 | Chr20:52781251 | G/C | 10(0.294) | 24(0.706) | 242(0.240) | 766(0.760) | 0.469 |
| CYP24A1 | rs6068816 | Chr20:52781091 | G/A | 15(0.441) | 19(0.559) | 391(0.388) | 617(0.612) | 0.53 |
| CYP24A1 | rs2296240 | Chr 20:5277572 | A/G | 23(0.676) | 11(0.324) | 610(0.605) | 398(0.395) | 0.402 |
| CYP24A1 | rs2296239 | Chr20:52775528 | C/T | 23(0.676) | 11(0.324) | 610(0.605) | 398(0.395) | 0.402 |
| CYP24A1 | rs2274130 | Chr20:52774601 | A/G | 23(0.676) | 11(0.324) | 610(0.605) | 398(0.395) | 0.402 |
| CYP24A1 | rs1977297 | Chr20:52774479 | C/T | 22(0.647) | 12(0.353) | 698(0.692) | 310(0.308) | 0.573 |

**S2 Table.** Association between CYP24A1 single nucleic acid gene polymorphism and the risk of colorectal cancer

| Gene | SNP | Position | Change | Cases(n=57) | | Controls(n=504) | | *P* |
| --- | --- | --- | --- | --- | --- | --- | --- | --- |
|  |  |  |  | Minor allele | Major allele | Minor  allele | Major  allele |  |
| CYP24A1 | rs6013905 | Chr20:52781264 | A/G | 40(0.351) | 74(0.649) | 410(0.407) | 598(0.593) | 0.249 |
| CYP24A1 | rs2762939 | Chr20:52781251 | G/C | 35(0.307) | 79(0.693) | 242(0.240) | 766(0.760) | 0.116 |
| CYP24A1 | rs6068816 | Chr20:52781091 | G/A | 40(0.351) | 74(0.649) | 391(0.388) | 617(0.612) | 0.441 |
| CYP24A1 | rs2296240 | Chr 20:5277572 | A/G | 62(0.544) | 52(0.456) | 610(0.605) | 398(0.395) | 0.206 |
| CYP24A1 | rs2296239 | Chr 20:52775528 | C/T | 62(0.544) | 52(0.456) | 610(0.605) | 398(0.395) | 0.206 |
| CYP24A1 | rs2274130 | Chr20:52774601 | A/G | 62(0.544) | 52(0.456) | 610(0.605) | 398(0.395) | 0.206 |
| CYP24A1 | rs1977297 | Chr20:52774479 | C/T | 22(0.647) | 12(0.353) | 698(0.692) | 310(0.308) | 0.328 |

**S3 Table.** Stratified analysis of the relationship between CYP24A1 polymorphisms and CRC as well as its risk factors by case-control

|  | rs6013905 | | | rs2762939 | | | rs6068816 | | |
| --- | --- | --- | --- | --- | --- | --- | --- | --- | --- |
| Subgroups | AA | GA | GG | CC | CG | GG | GG | AG | AA |
| **Age (years)** | | | | | | | | | |
| ≤60 | | | | | | | | | |
| Cases/Controls | 29(46.0)/  39(40.2) | 29(46.0)/  47(48.5) | 5(7.9)/  11(11.3) | 30(47.6)/  46(47.4) | 29(46.0)/  42(43.3) | 4(6.3)/  9(9.3) | 29(46.0)/  40(41.2) | 29(46.0)/  40(41.2) | 5(7.9)/  9(9.3) |
| OR(95% CI) |  | 1.21  (0.62-2.35) | 1.636  (0.51-5.22) |  | 0.945  (0.49-1.82) | 1.467  (0.41-5.20) |  | 1.200  (0.62-2.33) | 1.305  (0.40-4.30) |
| *P-*value |  | 0.583 | 0.403 |  | 0.865 | 0.551 |  | 0.59 | 0.661 |
| >60 | | | | | | | | | |
| Cases/Controls | 46(43.8)/  39(35.8) | 44(41.9)/  50(45.9) | 15(14.3)/  20(18.3) | 50(47.6)/  57(52.3) | 47(44.8)/  48(44.0) | 8(7.6)/  4(3.7) | 47(44.8)/  41(37.6) | 43(41.0)/  49(17.4) | 15(14.3)/  19(17.4) |
| OR(95% CI) |  | 1.340  (0.74-2.4) | 1.573  (0.71-3.48) |  | 0.896  (0.52-1.56) | 0.438  (0.12-1.54) |  | 1.306  (0.73-2.35) | 1.452  (0.66-3.22) |
| *P-*value |  | 0.329 | 0.262 |  | 0.67 | 0.19 |  | 0.371 | 0.357 |
| **Sex** | | | | | | | | | |
| Male | | | | | | | | | |
| Cases/Controls | 43(43.9)/  50(44.6) | 42(42.9)/  52(46.4) | 13(13.3)/  10(8.9) | 53(54.1)/  47(42.0) | 37(37.8)/  56(50.0) | 8(8.2)/  9(8.0) | 44(44.9)/  52(46.4) | 41(41.8)/  51(45.5) | 13(13.3)/  9(8.0) |
| OR(95% CI) |  | 1.064  (0.60-1.90) | 0.662  (0.26-1.66) |  | 1.707  (0.96-3.023) | 1.268  (0.45-3.55) |  | 1.053  (0.59-1.87) | 0.586  (0.23-1.50) |
| *P-*value |  | 0.831 | 0.377 |  | 0.066 | 0.65 |  | 0.861 | 0.262 |
| Female | | | | | | | | | |
| Cases/Controls | 32(45.7)/  28(29.8) | 31(44.3)/  45(47.9) | 7(10.0)/  21(22.3) | 27(38.6)/  56(59.6) | 39(55.7)/  34(36.2) | 4(5.7)/  4(4.3) | 32(45.7)/  29(30.9) | 31(44.3)/  46(48.9) | 7(10.0)/  19(20.2) |
| OR(95% CI) |  | 1.65  (0.84-3.28) | 3.42  (1.27-9.27) |  | 0.42  (0.22-0.81) | 0.48  (0.11-2.08) |  | 1.63  (0.83-3.22) | 3.00  (1.10-8.16) |
| *P-*value |  | 0.15 | 0.01 |  | 0.01 | 0.32 |  | 0.15 | 0.03 |
| **Occupation** | | | | | | | | | |
| Colon | | | | | | | | | |
| Cases/Controls | 27(37.5)/  78(37.9) | 37(51.4))/  97(47.1) | 8(11.1)/  31(15.0) | 35(48.6)/  103(50.0) | 33(45.8)/  90(43.7) | 4(5.6)/  13(6.3) | 27(37.5)/  81(39.3) | 37(51.4)/  97(47.1) | 8(11.1) /  28(13.6) |
| OR (95% CI) |  | 0.91  (0.51-1.62) | 1.34  (0.55-3.27) |  | 0.92  (0.53-1.61) | 1.11  (0.34-3.61) |  | 0.87  (0.49-1.56) | 1.16  (0.48-2.86) |
| *P-*value |  | 0.74 | 0.51 |  | 0.78 | 0.86 |  | 0.64 | 0.73 |
| Rectal | | | | | | | | | |
| Cases/Controls | 48(50.0)/  78(37.9) | 36(37.5)/  97(47.1) | 12(12.5)/  31(15.0) | 45(46.9)/  103(50.0) | 43(44.8)/  90(43.7) | 8(8.3)/  13(6.3) | 49(51.0)/  81(39.3) | 35(36.5)/  97(47.1) | 12(12.5)/  28(13.6) |
| OR (95% CI) |  | 1.531  (0.91-2.57) | 1.590  (0.75-3.39) |  | 0.91  (0.55-1.51) | 0.71  (0.28-1.83) |  | 1.67  (0.99-2.83) | 1.41  (0.66-3.03) |
| *P-*value |  | 0.11 | 0.22 |  | 0.72 | 0.48 |  | 0.05 | 0.37 |

**S4 Table.** Hardy - Weinberg of CYP24A1 SNP locus genotypes in the control group

| Genotype | actual | Expected | Gene | c2 | P |
| --- | --- | --- | --- | --- | --- |
| rs6013905 | |  |  | 0.391 | 0.532 |
| GG | 259 | 255 | 0.36 |  |  |
| GA | 333 | 341 | 0.47 |  |  |
| AA | 118 | 114 | 0.17 |  |  |
| rs2762939 | |  |  | 0.139 | 0.709 |
| GG | 399 | 397.12 | 0.56 |  |  |
| GC | 264 | 267.74 | 0.37 |  |  |
| CC | 47 | 45.13 | 0.07 |  |  |
| rs6068816 | |  |  | 0.364 | 0.547 |
| GG | 274 | 270.2 | 0.38 |  |  |
| AG | 328 | 335.59 | 0.46 |  |  |
| AA | 108 | 104.2 | 0.15 |  |  |

**S5 Table.** All primer sequences of CYP24A1

|  | Primer 5’-3’ |
| --- | --- |
| Exon 1F | ACATGGAGAGGGACAGGAGGAA |
| Exon 1R | GAGAGTCAGGGGCGCGAA |
| Exon 2F | CCCTGACTCTCTCCTCCCTCTT |
| Exon 2R | TGTACAAGAGCTCAGGGTTGCGA |
| Exon 3F | TGCCCCCTTTTACCGCTAGGA |
| Exon 3R | CCCCCACTTTGAATCACCCGA |
| Exon 4F | AGGGACAGTTAGCTGATTCCTTT |
| Exon 4R | GCCTTTCCCTAGGCAGCAATAA |
| Exon 5F | ACACAGCTGGGTTAGAGGCAGA |
| Exon 5R | AAAGGTTGGAGACAGCCGCAT |
| Exon 6F | ACACATCCAGTGGAAAATCATCCC |
| Exon 6R | ATTTGTGTATGCTGGGGCAATC |
| Exon 7F | GTATTGAAAAATCCTTGCCGACTTG |
| Exon 7R | GTTTAAGCACAGAAGCCCGT |
| Exon 8F | CGTAGCTATAAGGACGCGTGA |
| Exon 8R | GAGGGCACTGGCTTTCCAA |
| Exon 9F | CAGCCTTGCACTGGGGTTT |
| Exon 9R | TCTCTGTCTCCATAGCCGTGA |
| Exon 10F | GTGAGCCTCAACTTCGTCTGT |
| Exon 10R | TGAGGCGTATTATCGCTGGCAA |
| Exon 11F | ACACCACACCATACAACAGCTT |
| Exon 11R | TTTCAATGCAGGAAGAACGCAA |
| Exon 12F | TAGGAAAATGTCCCTGCTTA |
| Exon 12R | GCTTGTAGGCCATATGAGAA |
